# Cross-relationship between COVID-19 infection and anti-obesity products efficacy and incidence of side effects: A cross-sectional study

**DOI:** 10.1371/journal.pone.0309323

**Published:** 2024-08-22

**Authors:** Nesrine S. El-Mezayen, Yasser R. Abelrazik, Dina M. Khalifa, Nada M. Dorbouk, Mai A. Moaaz, Merna M. Ali, Alaa G. Evy, Elshaimaa G. Mohamed, Ahmed M. Abdelhadi, Irinie Adly, Nilly A. Shams

**Affiliations:** 1 Faculty of Pharmacy, Department of Pharmacology and Therapeutics, Pharos University in Alexandria, Alexandria, Egypt; 2 Faculty of Pharmacy, Phftablearos University in Alexandria, Alexandria, Egypt; 3 Dr. Nilly Shams’ Nutrition Center, Alexandria, Egypt; Universiti Monash Malaysia: Monash University Malaysia, MALAYSIA

## Abstract

**Background:**

Obesity and COVID-19 are at the top of nowadays health concerns with significant crosstalk between each other. The COVID-19 pandemic negatively affected healthy lifestyles and increased obesity prevalence. Thus, there was a surge in anti-obesity products (AOPs) intake. Herein, we evaluated how the pandemic has affected slimming products’ efficacy and safety in patients seeking weight reduction at an urban, weight management centre in Alexandria, Egypt. In addition, the effect of AOPs on COVID-19 infection severity was also appraised to detect whether AOPs can alter COVID-19 host cell entry and infective mechanisms, and thus, affect infection severity.

**Methods:**

Patients were invited to complete an anonymous survey. The survey assessed self-reported changes in weight, the use of AOPs during the COVID-19 pandemic, COVID-19 infection severity, AOPs efficacy, and incidence of side effects. Inclusion criteria were obese patients above 18 years old who got infected by COVID-19 while receiving a single-ingredient AOP.

**Results:**

A total of 462 participants completed our anonymous validated questionnaire. Most of the participants were females (450; 98.4%) with BMI ranging from 24.98–58.46. Eligible participants were only 234 and the top-administered products were orlistat, liraglutide, metformin, green tea, cinnamon, *Garcinia cambogia*, and *Gymnema Sylvestre*. In most cases, AOPs intake was beneficial for COVID-19 infection, and most patients experienced mild-to-moderate COVID-19 symptoms. On the other hand, SARS-CoV-2 significantly interferes with AOPs’ mechanisms of action which positively or negatively influences their efficacy and side effects incidence due to predictable pharmacological link.

**Conclusion:**

Concurrent AOPs intake with COVID-19 infection is a two-sided weapon; AOPs attenuate COVID-19 infection, while SARS-CoV-2 interferes with efficacy and side effects incidence of AOPs.

## 1. Background

Nowadays, overweight and obesity are among global epidemics comprising more than 20%overweight/obesity prevalence [1]. A dramatic rise in overweight/obesity cases was detected in low- and middle-income countries, predominantly in urban sectors, representing major risk factors for myriad chronic illnesses. According to the World Health Organization (WHO), obesity is defined as an excessive abnormal fat accumulation that poses health risks. Overweight individuals have a body-mass index (BMI)>25, and those>30 are considered obese [2]. The pathogenesis of obesity implicates dysregulation of balance between consumed and expended calories, physical activity, and appetite control. All of these are controlled by leptin, insulin hormones, and hypothalamic neurons through a complex interplay with hereditary, environmental factors, and intestinal microbiome [2].

The COVID-19 pandemic caused by the novel coronavirus SARS-CoV-2 seriously affected billions of people around the globe for >two years. COVID-19 has claimed over 5.6 lives and has caused all countries worldwide to set policies to implement social distancing and restrict movement to impede viral spreading. These amendments have caused alterations in food consumption and patterns of physical activity. In addition, remote telework environments were created that exacerbated the prevalence of obese individuals [1]. These alterations resulted in long-lasting implications on people’s health beyond SARS-CoV-2 spread mitigation.

A strong association between obesity and COVID-19 infection has been demonstrated [1,3,4]. Obesity increases the vulnerability to COVID-19 infection as both diseases share common pathologic pathways. Indeed, the Centers-for-Disease-Control-and-Prevention (CDC) specified that individuals with a BMI≥40 are at a higher risk for severe COVID-19 illness [5,6].

Furthermore, obesity leads to poor prognosis in patients with SARS-CoV-2 represents a state of chronic low-grade inflammation. This is because the hyperplastic or hypertrophied adipose tissues secret various inflammatory products and cytokines causing a hyperinflammatory response leading to hypoxia and ischemia, which results in an oxidative stress state involving the release of inflammatory proteins and reactive oxygen species that impair the mitochondrial function worsening COVID-19 prognosis [3]. Moreover, obesity significantly decreases the effectiveness of COVID-19 vaccines and increases morbidity and mortality from COVID-19 [1]. Therefore, there are recommendations that individuals with obesity should closely follow the clinical guidelines for obesity management and that healthcare providers should ensure that these patients do not stop taking antiobesity agents during the COVID-19 pandemic [3].

Several studies have analyzed the correlation between angiotensin-converting enzyme-2 (ACE2) and COVID-19. ACE2 is a part of the counter-regulatory renin-angiotensin system that converts angiotensin (Ang)-II to Ang-(1–7). Although the ACE2/Ang (1–7) system exerts anti-inflammatory/anti-oxidant functions that protect the lung against respiratory distress, SARS-Cov-2 can utilize ACE2 receptors for host cell entry. Of note, ACE2 expression can be altered by several factors, including obesity, which could further increase COVID-19-infection severity [5]. Cumulative evidence from *in-silico* and *in-vitro* studies pointed to increased ACE2 expression, mostly in the lungs, in obese subjects due to SARS-Cov-2-induced lipid metabolism dysregulation. Increased ACE2 expression enhances ACE2 receptor-mediated SARS-CoV-2 viral entry. Subsequently, the dysregulated lipid metabolism increases sterol regulatory element binding proteins, which in turn suppresses ACE2. ACE2 suppression is the main driver for COVID-19-coupled lipotoxicity and inflammation. Thus, ACE2 exerts a dual action during COVID-19 infection; it facilitates viral entry and participates in the demonstrated COVID-19-associated lipid metabolism dysregulation, a key obesity feature [7].

The increased obesity prevalence during the COVID-19 pandemic, enthused a surge in following dietary restrictions, or more effortlessly, consuming weight reduction products with or without medical consultation. FDA-approved anti-obesity pharmacotherapy is recommended for those whose BMI is ≥30, or ≥27 with comorbid conditions, or those incapable of losing weight using lifestyle modification alone. There are only four FDA-approved anti-obesity drugs for short-term obesity treatment including; orlistat, liraglutide, naltrexone-bupropion, and phentermine-topiramate, in addition to gelesis which is approved for long-term obesity control [8]. Due to the growing go-green and returning to nature affinity for fear of adverse effects from approved prescribed medications, people are progressively administering herbal preparations. The natural origin of these non-approved herbal products provides users with a sense of security and encourages long-term use. However, most herbal anti-obesity-products (AOP) are adulterated with synthetic illegally-added compounds with significant toxicity, among them are phentermine, sibutramine, amphetamines, bupropion, phenolphthalein, phenytoin and bumetanide [9]. So far, the impact of COVID-19 on the safety and efficacy of the highly utilized approved and non-approved AOP during the pandemic and the back-influence of these products on the severity of COVID-19 symptoms seems to be under-investigated.

Though several studies have been published evaluating the impact of COVID-19 on many disorders including overweight and obesity, little is known about how COVID-19 is influencing the surge for using prescribed as well as non-prescribed AOP and whether COVID-19 has an impact on their efficacy and side-effects severity. Further, it is unclear whether AOP can affect mechanisms used by COVID-19 for host cell entry and infection severity. One study conducted by De la Rosa et al revealed that patients taking FDA-approved AOP post-COVID-19 infection have lost significantly less body weight percentage compared to the pre-COVID-19 group. Moreover, some patients in that study discontinued AOP intake due to reported incidence of side effects [10]. For this purpose, we evaluated how the pandemic has affected AOP efficacy and the incidence of side effects in patients seeking weight reduction at a large, urban, weight management center (Dr. Nilly Shams’ Nutrition Center (NSNC) in Alexandria, Egypt using a standardized questionnaire. In addition, the effect of AOP on COVID-19 infection severity was also appraised. To the best of our knowledge, we present the first study that investigates the possibility of interactions between COVID-19 and AOP.

## 2. Methods

### 2.1. Study design and eligibility criteria

Patients≥ 18 years old seeking weight reduction in the period between 1^st^ March/2020-1^st^March/2022 at NSNC for nutritional weight control in Alexandria, Egypt were invited to complete an anonymous survey. The survey includes some demographic data and the medical history of the patients. It assessed self-reported changes in weight and the use of AOPs following the issuance of social distancing/stay-at-home policies in 2020.

Inclusion criteria for statistical analysis were being infected by COVID-19 while receiving the AOPs and receiving single-ingredient AOPs. The survey assessed the changes in medication efficacy and the incidence of side effects following COVID-19 infection. Further, the severity of COVID-19 symptoms during and after AOP intake was also evaluated.

### 2.2. Sample size calculation

Sample size calculation for cross-sectional studies/surveys was calculated according to Eq (1) for pursuing scientific rigor [11]. Where Z is the confidence level equals 1.96 for 95% confidence, P is the expected prevalence based on a pilot study (equals 42%), and d is the degree of precision (margin of error) (estimated to be 5%-6%). The calculated sample size is 374 or 256 for d values 0.05 and 0.06, respectively.


$${Sample\;size=Z}^{2}\;x\;P(1-P)/d^{2}$$ (1)

### 2.3. Ethical approval

The study was approved by the Pharos-University-in-Alexandria (PUA) Research-Ethics-Committee, Alexandria, Egypt following STROBE guidelines. The ethics committee waived the need for informed consent.

### 2.4. Development of the questionnaire

The process of developing and validating a questionnaire followed a Standardized methodology that comprised multiple steps; literature review, focus group discussion, pilot study (pretesting), validation of the questionnaire, etc. Questionnaire generation comprised the following steps:

#### 2.4.1. Questionnaire-generation

A literature review was done using PubMed and other medical search engines to seek research done over the past three years. Based on this comprehensive research, questions were generated from formerly related questionnaires. The literature review was followed by qualitative data analysis and other questions and items were added. Survey items were written in Arabic language, which could be understood by all the participants.

**Fig 1:**
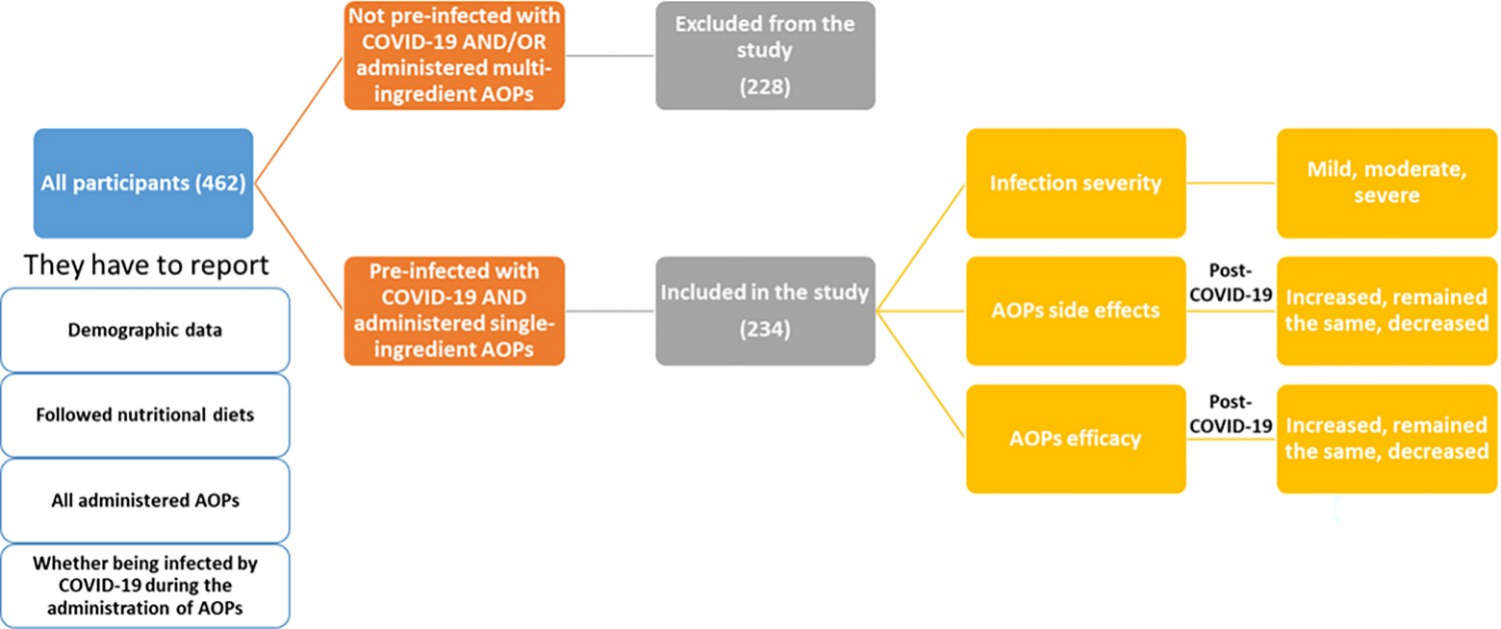
Summary of the main gathered information in the developed questionnaire. Demographic data (age, sex, weight & height (for calculating BMI), underlying health conditions).

The developed questionnaire is presented as a (S1 File) and it generally gathered the following information (Fig 1):

- Demographic data (age, sex, weight & height (for calculating BMI), underlying health conditions…).
- Nutritional diets followed by participants.
- All administered AOPs (their side-effects and efficacy (estimated by weight reduction/week)).
- Whether being infected by COVID-19 during the administration of AOPs.
- Severity of COVID-19 infection (triple scale: mild, moderate, and severe).
- Whether they continued using AOPs post-COVID-19 infection.
- AOPs side-effects post-COVID-19-infection (triple scale: increased, remained the same, decreased). They had to mention absent or new side effects as well.
- AOP efficacy post-COVID-19 infection (estimated by weight reduction/week) (triple scale: increased, remained the same, decreased).

#### 2.4.2. Questionnaire-validation by experts

The questionnaire was validated by six experts who critically and independently validated all questionnaire items following the procedure described by Olson et al [12]. For each question, the reviewers were asked to rate the cognitive characteristics (possibility of failure of the cognitive response process for a question), and motivational characteristics (whether the question was burdensome, sensitive, or socially undesirable). For questions rated as possibly experiencing failures, each stage of the cognitive response process (comprehension, retrieval, judgment, and editing) was rated on a 4-point scale ranging from not at all likely to very likely that a failure of this stage will occur. Experts then provided commentaries about the problems they believed would happen.

#### 2.4.3. Pilot study and questionnaire validation

The pilot study aims to detect any ambiguity in the participant’s comprehension of the generated questions. The final questionnaire was posted to 30 participants and minor changes were made to the questionnaire as per their comments. After that, a survey was performed for questionnaire validation.

In addition, a reliability analysis (Cronbach’s alpha) test was done for the final questionnaire to measure the internal consistency of survey items. Cronbach’s alpha test result was 0.912 indicating high and suitable reliability indices.

#### 2.4.4. Participants and survey procedure

The questionnaire was administered to a total of 462 participants at NSNC between 16^th^ March/2022 - 16^th^ June/2022. These participants were sorted according to the inclusion criteria and only data from eligible participants were subjected to further analysis.

### 2.5. Statistical analysis of the data

Data were fed to the computer and analyzed using the IBM-SPSS software package version 20.0**. (**Armonk, NY: IBM-Corp**).** Qualitative data were described using numbers and percentages. The significance of the obtained results was judged at the 5% level. The used tests were the Chi-square test; for categorical variables, to compare between different AOPs and Monte-Carlo correction; Correction for chi-square when>20%of the cells have expected count<5.

## 3. Results

### 3.1. Participants data

**Fig 2:**
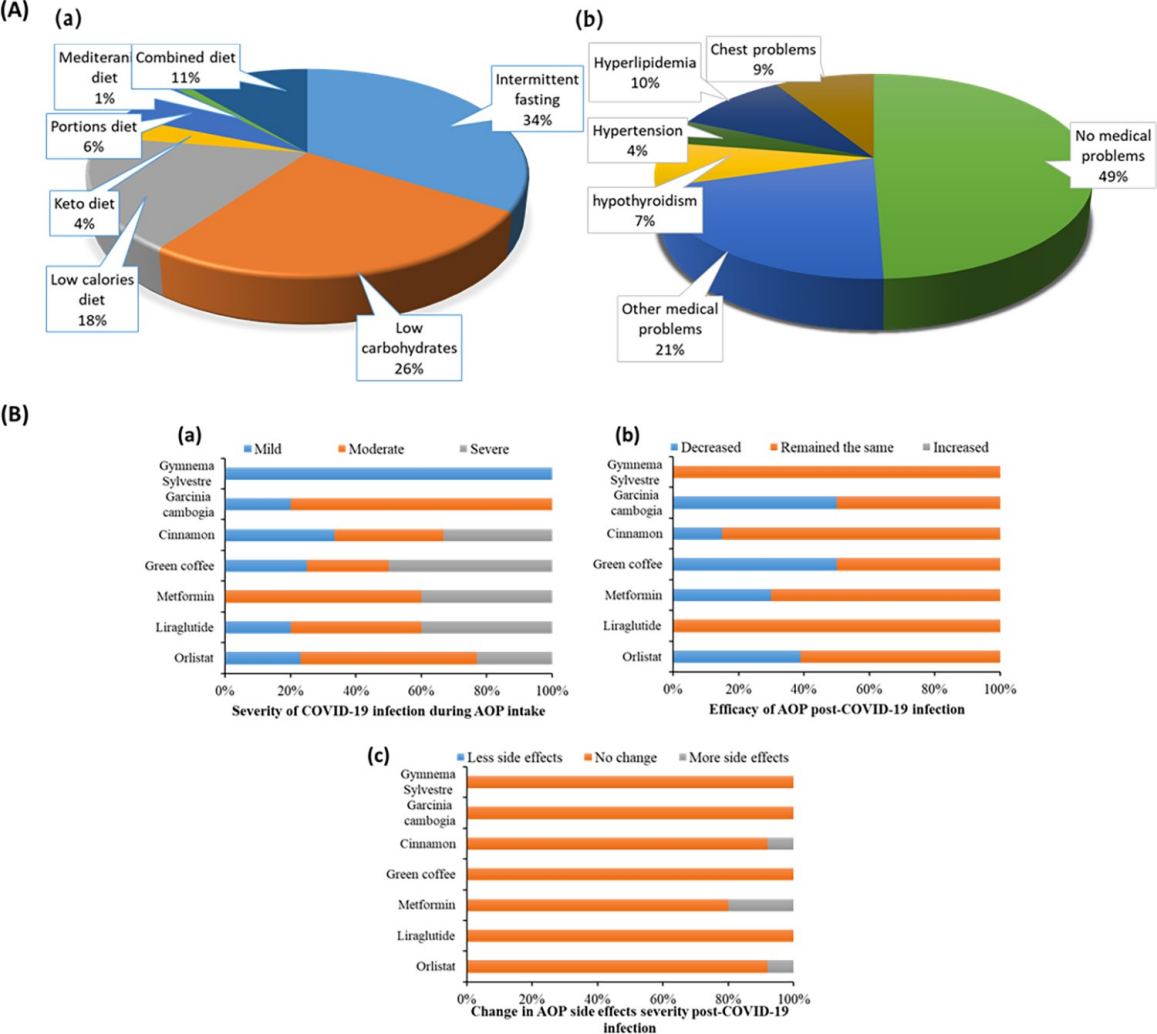
Participants’ nutritional diets/medical history and crosstalk between COVID-19 infection and AOPs. A: Nutritional diets followed by participants (a) and their reported medical history (b); B: 100% stack bar charts showing the impact of AOP on COVID-19 infection severity and influence of COVID-19 infection on AOP efficacy and incidence of side effects. (a: Reported severity of COVID-19 symptoms following AOP administration, b: Efficacy of AOP post-COVID-19 infection, c: AOP side effects severity post-COVID-19 infection. (AOP: Anti-obesity products. Products included in the table are the top utilized products by the survey-eligible participants. Eligible participants are those taking only single-ingredient products (n = 234)).

A total of 462 participants completed our anonymous questionnaire between 16^th^ March/2022-16^th^June/2022. Most of the participants were females (450;98.4%). The percentage of participants falling into different age categories (subdivided into decades) and their reported medical history is shown in Fig 2A. BMI for all participants ranged from 24.98 to 58.46. The BMI and medical history for each age range of participants are also presented in Table 1.

**Table 1:** The BMI and medical history for each age range of participants (n = 462).

Age range	% Participants	BMI (kg/m2)	Health problems
**15–20**	**5.84%**	**Mean±SD:** 33.5±6.2	81.48%: No health problems, 11.11%: anemia, 7.4%: hypothyroidism
		**Range:** 25.4–45.7	
**20–30**	**36.36%**	**Mean±SD:** 32.8±5.63	60.12%: no health problems, 6.55%: hyperlipidemia, 4.17%: respiratory problems, 2.38%: diabetes, 26.78%: other problems including psoriasis, osteoarthritis, polycystic ovary, etc..
		**Range:** 25.15–49.49	
**30–40**	**42.2%**	**Mean±SD:** 35.35±15.98	44.1%: no health problems, 12.82%: hypothyroidism, 11.28%: hyperlipidemia, 3.6%: hypertension, 2.56%: diabetes, other medical problems include autoimmune diseases, osteoarthritis, polycystic ovary, etc..
		**Range:** 24.98–58.46	
**40–50**	**13.65%**	**Mean±SD:** 34.83±6.21	24%: no health problems, 10%: osteoarthritis, 8%: hyperlipidemia, 7%: hypothyroidism, 4%: hypertension, 1%: diabetes, other medical illnesses include: depression, benign and malignant tumors, chest problems
		**Range:** 25.63–54.69	
**>50**	**1.06%**	**Mean±SD:** 34.79±4.57	20%: hyperlipidemia, 20%: hypertension, 20%: dysrhythmia, 20%:diabetes, 20: mitral valve problems
		**Range:** 29.74–40.52	

### 3.2. Participants’ nutritional attitude and surge for weight reduction during the COVID-19-outbreak

All participants followed certain diets, with intermittent fasting being the most applied diet (34%) (Fig 2Aa), however, only 72.7% of them were committed to diet rules. Surprisingly, all participants administered AOP in all its forms whether herbal, non-prescription drugs or OTC-FDA-approved anti-obesity drugs with or without consultation from the health care provider. Of them, only 21.2% received anti-obesity FDA-approved medicines, and the remainder received herbs, with known weight reduction properties, and non-FDA-approved AOP. Most participants administered multi-ingredient preparations (The analysis of the influence of each single component was nearly impossible), and thus, their data was excluded from the study. Only 234 participants administered a single AOP and were eligible for further analysis. About 42%of these eligible participants got infected with COVID-19 during receiving AOP and their reported data was analyzed to find the inter-correlation between their used AOP and COVID-19-infection.

### 3.3. Cross-talk between FDA-approved medications & COVID-19-infection

#### 3.3.1. Orlistat

According to the present survey, 23.8%of eligible participants administered orlistat under different brand names. Of them, 66.67%reported an incidence of side effects. Most reported side-effects were as follows: GIT side-effects (51.8%), rebound increase in weight after discontinuing the drug (10%), fatigue (10.9%), urticaria (6.67), and CNS side-effects including; headache and depression (6.67%), Table 2. About 43.33%participants taking Orlistat got infected with COVID-19 during treatment. The infection was severe in 23.1%of these patients, mild in 23.1%, and moderate in 54.8%. After recovery from COVID-19, 61.5%patients continued using Orlistat. Most of them reported unaltered efficacy and 38.5% reported a decrease in orlistat efficacy in reducing weight. Remarkably, no change in the severity of orlistat side-effects was observed following infection with COVID-19, as only 7.7%reported increased severity of GIT symptoms (of note, those who reported severe COVID-19 symptoms were those reporting increased severity of GIT side-effects) (Fig 2B).

**Table 2:** Percentages of eligible participants receiving single AOP, reported side effects for each product, and percentage of participants that got infected with SARS-CoV-2 during AOP intake (n = 234).

AOP*	% Eligible participants** taking the drug relative to total eligible participants	Most reported side effects	% Participants that got infected with SARS-CoV-2 during the course of AOP intake
**Orlistat**	23.8%	GIT side effects (51.8%), fatigue (10.9%), rebound increase in weight after discontinuing the drug (10%), urticarial (6.67), CNS side effects including headache, depression (6.67%)	43.33%
**Liraglutide**	12.6%	Nausea (53.33%), hypoglycaemia (33.33%), dysrhythmia (6.67%), menstrual disturbance (6.67)	80%
**Metformin**	12.2%	Hypoglycaemia (66.67%), nausea (50%), dysrhythmia (16.67%)	71%
**Green coffee**	10.8%	Headache (11.11%)	45%
**Cinnamon**	8.8%	GIT disturbance (13.3%), hypertension (6.7%)	60.00%
**Garcinia cambogia**	6%	Diarrhea (10%)	44%
**Gymnema Sylvestre**	1.2%	----	50%

*AOP: Anti-obesity products. Products included in the table are the top utilized products by the survey participants.

** Eligible participants are those taking only single-ingredient AOPs.

#### 3.3.2. GLP-1-receptor-agonists (GLP-RA)

12.6%participants were administering GLP-RA (liraglutide), Table 2. Among them, 51.7%experienced side effects including; nausea (53.33%), hypoglycemia (33.33%), dysrhythmia (6.67%), and menstrual disturbance(6.67), Table 2. Eighty % of participants taking liraglutide caught COVID-19 infection while on therapy. The infection was moderate-to-severe in nearly 80% of these patients and mild only in 20%. Notably, patients experiencing severe COVID-19 symptoms were extremely obese with BMI >35. Post-COVID-19, only 20%of patients continued using liraglutide and they reported no influence of the infection on either efficacy or side-effects incidence of the administered drug (Fig 2B).

#### 3.3.3. Other FDA-approved AOPs

There was no single case in this study where participants reported the use of any FDA-approved AOPs other than orlistat or liraglutide.

### 3.4. Cross-talk between non-FDA-approved slimming products &COVID-19

In the current survey, nearly 81% of participants were non-FDA-approved AOPs either drugs or herbal products without a physician’s prescription. The most used slimming agents were: metformin garcinia/chromium, green coffee, cinnamon, Coffea-Arabica, African-mango, Gymnema-Sylvestre, etc. In addition, a huge number of slimming products (mostly imported) claiming to contain a combination of the above-mentioned herbs were used by the majority of the participants.

During our data analysis, it was noticed that some participants who administered those imported multi-ingredient slimming products have reported unrelated side effects to the herbs enclosed in the product. The reported unrelated side effects include nervousness, insomnia (one participant reported not being able to sleep for three successive days), headache, palpitations, and hypertension.

#### 3.4.1. Metformin

Metformin was the third in order of the highly-administered single AOPs in the current survey. About 12.2%eligible participants were taking metformin and 42.9% of them reported an incidence of side effects. The most reported side effects were hypoglycemia (66.7%) and nausea (50%), Table 2. A high percentage of participants who administered metformin got infected with SARS-CoV-2 (71%). Around 60% of infected participants reported experiencing moderate symptoms and ≈40%reported experiencing severe COVID-19 symptoms. Nearly 94%of these patients continued using metformin after recovery from COVID-19 and 70%of participants reported no change in metformin efficacy and 30%reported decreased efficacy. A great percentage of these participants (80%) reported no change in the severity of metformin side effects post-COVID-19 and 20% reported an increase in the incidence of hypoglycemia (Fig 2B).

#### 3.4.2. Green-coffee

According to the data obtained from the survey, 10.8%of eligible participants were administering green coffee. About 11.11%reported the incidence of headache as a main side-effect and the remainder 88.99%reported the absence of side-effects, Table 2. Around 45.44%of these participants were infected with COVID-19 during green-coffee administration. The infection symptoms were mild in 25%, moderate in 25%, and severe in 50%. After recovery from COVID-19, 66.67% of these participants continued using green coffee 50% of them reported a decrease in green coffee efficacy in weight reduction and 50% reported no change in weight reduction efficacy. However, all participants who continued taking green coffee after the SARS-CoV-2-infection reported no change in green coffee side-effects severity (Fig 2B).

#### 3.4.3. Cinnamon

Cinnamon represented the second most administered single-ingredient herb representing 8.8%of total eligible participants. Only 20%of participants on cinnamon reported an incidence of side effects. The most reported side effects were GIT disturbance (13.3%) and hypertension (6.7%), Table 2. Amongst participants taking cinnamon around 60%were infected with COVID-19 during administration. The infection severity was mild in 37.5%, moderate in 30.7%, and severe in 30.8%. A large percentage of these participants (69.2%) continued using cinnamon after recovery from COVID-19. The majority (84.6%) reported no change in cinnamon weight reduction efficacy and 15.4%reported decreased cinnamon efficacy. Most participants (92.3%) stated that there was no change in the incidence of cinnamon side effects post-COVID-19-infection, whereas, 7.7% reported an incidence of new-onset GIT symptoms that were not noticed before COVID-19-infection (Fig 2B).

#### 3.4.4. Garcinia-cambogia

Though only 6%eligible participants were administering *Garcinia-cambogia* in its pure form, 48.3%of total participants were administering *Garcinia-cambogia*-containing multi-ingredient herbal products under different brand names. The upcoming analysis concerns the 6%that were administering pure *Garcinia-cambogia* for a fair evaluation of its impact on COVID-19-infection severity and the influence of infection on *Garcinia-cambogia* efficacy and side-effects incidence. Nearly 90%of participants did not experience any detectable side effects and only 10% reported an incidence of diarrhea; Table 2. <50%of participants taking pure *Garcinia-cambogia* (⁓44%) got infected with COVID-19 during Garcinia intake with mild symptoms in20% and moderate symptoms in 80%. Nearly 60%of participants continued using *Garcinia-cambogia* after recovery from COVID-19. Exactly half of them reported maintaining similar efficacy after infection and the other half reported decreased efficacy. Remarkably, no change in side-effects severity was observed following infection with COVID-19 in all participants who continued taking *Garcinia-cambogia* post-COVID-19 infection (Fig 2B).

#### 3.4.5. Gymnema-Sylvestre

A few percent of participants were taking *Gymnema-Sylvestre* (*G*.*Sylvestre*) as a single-ingredient(1.2%) and all of them negated the incidence of any side-effects, Table 2. Half of these had mild COVID-19 infection, and the other half were not infected. All participants that continued using *G*.*Sylvestre* post-COVID-19 infection (30%) were not regularly taking the herb and all negates change in *G*.*Sylvestre* efficacy or side-effects incidence (Fig 2B).

### 3.5. Comparison between the top-administered AOP according to the severity of COVID-19-infection during AOP intake

The greatest incidence of severe COVID-19 infection and symptoms was observed in patients administering green coffee, followed by liraglutide, metformin, and cinnamon with no statistically significant difference between them. Participants on either *Garcinia-cambogia* or *Gymnema-Sylvestre* who got infected with COVID-19 did not report the incidence of any severe symptoms. Reporting experiencing moderate COVID-19 symptoms was the most common incidence among participants taking AOPs, S1 Table.

### 3.6. Comparison between the top-administered AOP according to AOP efficacy and incidence of side-effects post-COVID-19-infection

No single participant reported increased efficacy of any administered AOP post-COVID-19 infection. They mostly reported decreased efficacy of the administered AOPs in reducing weight, except for liraglutide and *Gymnema-Sylvestre* where patients reported maintaining their efficacy post-COVID-19-infection, S2 Table. Likewise, participants mostly reported no change in the incidence of AOP side-effects post-COVID-19-infection for the top-administered AOPs in our survey. However, an increased incidence of AOP side effects post-COVID-19 was reported in some participants administering metformin, orlistat, and cinnamon. Yet, at the statistical level, the increased incidence of side effects reached a significance level only for metformin, S3 Table.

## 4. Discussion

The COVID-19 pandemic seriously affected billions of people around the globe for more than two years. COVID-19 has caused all people to restrict movement and caused alterations in food consumption and physical activity patterns. Consequently, obesity prevalence has increased during the pandemic and there was a great surge in following dietary restrictions and consuming AOP even without medical consultation. Herein, we aimed to investigate the impact of COVID-19 on the safety and efficacy of the highly utilized AOP during the pandemic in patients seeking weight reduction at NSNC using a standardized questionnaire. In addition, the influence of these AOPs on COVID-19 infection severity was also appraised.

In general, participants reported decreased efficacy of the administered AOPs in reducing weight, except for liraglutide and *Gymnema-Sylvestre* where patients reported maintaining their efficacy post-COVID-19 infection. In accordance with our results, a study performed by De la Rosa et al. [10], a retrospective systematic review of electronic medical records and included patients who started a long-term FDA-approved AOP, reported significantly inferior weight loss outcomes during the routine clinical practice throughout COVID-19 pandemic compared to the outcomes observed before the COVID-19 pandemic [10]. An increased incidence of AOP side effects post-COVID-19 was reported in some participants administering metformin, orlistat, and cinnamon which reached a significance level only for metformin. In the study of De la Rosa et al. [10], the authors reported that some participants discontinued the study due to the incidence of side effects without specifying either the administered AOPs for these participants or their reported side effects.

### 4.1. Orlistat

Orlistat was the most used AOP by the participants and 23.8%of eligible participants (taking single-ingredient products) were administering orlistat under different brand names. The most reported side-effects with Orlistat were the GIT side-effects (51.8%), which is per Orlistat documented side-effects [13].

About 43%of participants taking orlistat got infected with COVID-19 and most of them (77.9%) experienced mild-to-moderate infection symptoms, reflecting a possible protective effect exerted by orlistat. This is because orlistat inhibits cellular lipid synthesis which is crucial for SARS-CoV-2 replication. In support of this result, it was previously proved that fatty acid synthase inhibitors, including orlistat, inhibit *in-vitro* replication of SARS-CoV-2 variants, even the more contagious new variants, such as Delta [14]. In a mouse model of SARS-CoV-2-infection (K18-hACE2 transgenic mice), orlistat injections lowered SARS-CoV-2 viral levels in the lung, reduced lung pathology, and increased mouse survival [15].

After recovery from COVID-19, 61.5%of the patients continued using orlistat. Most of them reported unaltered efficacy and 38.5% reported a decrease in orlistat efficacy in reducing weight. Remarkably, no change in the severity of orlistat side-effects was observed following infection with COVID-19, as only 7.7% reported increased severity of GIT symptoms (of note, those who reported severe COVID-19 symptoms were those reporting increased severity of GIT side-effects.

**Fig 3:**
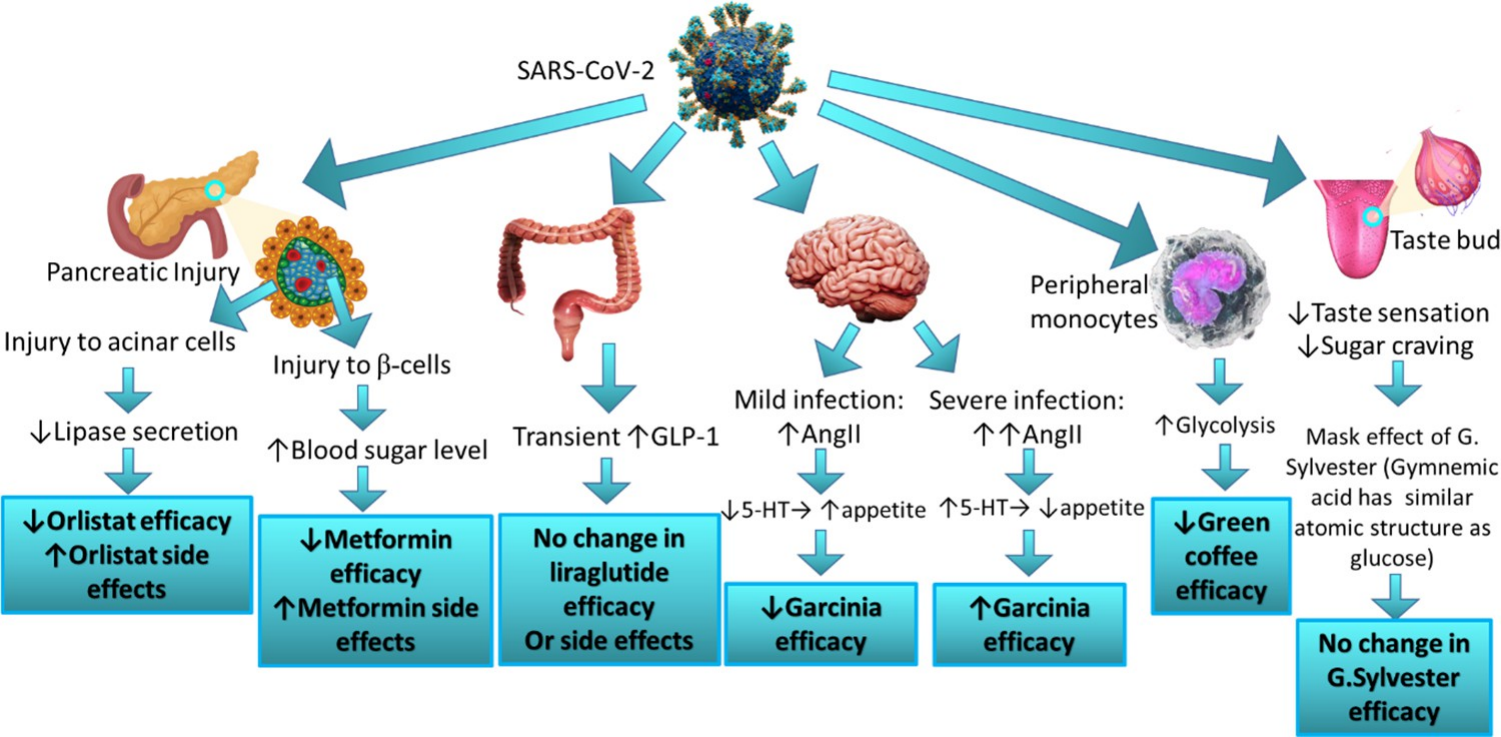
The possible influence of COVID-19 infection on weight reduction mechanisms of top-administered AOP in the present study.

ACE2, required for SARS-CoV-2 host entry, is expressed in the pancreas of normal people. This expression is slightly higher in the pancreas than in the lungs, indicating that SARS-CoV-2 also might bind to ACE2 in the pancreas and cause pancreatic injury [16]. Furthermore, single-cell RNA sequencing data indicated that ACE2 is expressed in both exocrine glands (secreting lipase which is the molecular target of orlistat) as well as islets of the pancreas. This means that COVID-19 may impact orlistat efficacy and side effects if the infection causes pancreatic damage (Fig 3). This pancreatic injury may cause a decrease in lipase production which may cause additive side effects to those experienced by lipase inhibition by orlistat. Since one cohort study showed that approximately 1–2%of non-severe and 17%of severe patients with COVID-19 had a pancreatic injury [17], additive orlistat side-effects may be more visible in patients with severe COVID-19-infection and this perfectly matched our results.

### 4.2. GLP-1-receptor-agonists (GLP-RA)

GLP-1 is released by the endocrine L-cells in the distal intestinal mucosa in response to the presence of nutrients in the intestinal lumen. Once in circulation, GLP-1 has a short half-life due to fast breakdown by the enzyme dipeptidyl-peptidase-IV(DPP-IV). GLP-1 causes insulin release from pancreatic beta-cells after eating, reduces glucagon secretion, and slows gastric emptying leading to a fall in blood glucose levels [18]. GLP-1 also acts peripherally and centrally to affect food intake and metabolism by regulating circulating levels of leptin [19].

Liraglutide, a GLP-RA, shares 97%structural-homology with human GLP-1 but has a prolonged half-life (10–14hrs). Liraglutide improves glycemic control and induces body weight loss with an acceptable limit of side effects [20]. In the current survey, 12.6% of participants administered liraglutide, and nearly half experienced side effects. The most reported side-effects were nausea, hypoglycemia, and dysrhythmia which is in agreement with the documented side-effects [21].

While on liraglutide therapy, 80% of participants got infected with SARS-CoV-2. The infection was moderate-to-severe in nearly 80% of these patients and mild only in 20%. Notably, patients experiencing severe COVID-19 symptoms were extremely obese with BMI>35. In accordance, many studies documented the beneficial effects of GLP-1-RA in protection against/treatment of SARS-CoV-2 infection. Reports indicate that liraglutide and semaglutide, as GLP-1-RA, can reduce inflammation-induced lung injury. They are considered excellent candidates for treating COVID-19 patients with or without type-2-Diabetes-Mellitus [22]. GLP-1-RA can probably exert a pulmonary protective effect rather than modulating SARS-CoV-2 infection. During COVID-19 viral infection, suppression of the protective arm of the renin-angiotensin-system (RAS) occurs leading to a significant reduction in ACE2 activity. This directs the RAS towards stimulating the pro-inflammatory arm by increasing ACE activity, A disintegrin, metalloprotease-17 activation, and overproduction of pro-inflammatory cytokines. This in turn leads to a pro-thrombotic milieu and “cytokine storm” exacerbation in response to SARS-CoV-2-infection [23]. The ability of GLP-1-RA to significantly increase gene expression of RAS protective arm components; ACE2/Ang (1–7)/Mas-receptor at the pulmonary level was previously proved [24]. Consequently, GLP-1-RA can enhance the production of prostacyclin and nitric-oxide levels, and reduce angiotensin-II-type-1-receptor (AT1R) activation which may counteract the negative consequences of SARS-CoV-2-infection. As a support, GLP-1-RA has well-documented cardiovascular protective effects, however, more investigations are needed to validate ACE2’s role in mediating the beneficial multi-organ extraglycemic effects of this class of medication [23].

In the present study, patients declared the absence of influence of COVID-19 infection on the efficacy or side-effects incidence of liraglutide. A possible explanation will be given away in the next few lines; SARS-CoV-2-induced RAS activation leads to increased colonic production of GLP-1. This is mainly because AT1R is highly expressed in enteroendocrine L-cells, that produce GLP-1 and stimulate its release [25]. This may add benefit to the observed efficacy of liraglutide. However, since the endogenously secreted GLP-1 has a half-life of <2 minutes once in circulation, SARS-CoV-2-induced production of GLP-1 may not produce an observable influence on GLP-1-mediated effects (Fig 3).

### 4.3. Other FDA-approved AOPs

Although there were no participants in the present study that reported the use of any FDA-approved AOPs other than orlistat or liraglutide, a prediction about the expected outcomes, if patients were administered topiramate/phentermine or naltrexone/bupropion, was made. This is because, during data analysis, it was noticed that some non-eligible participants who administered multi-ingredient AOPs reported unrelated side effects to the herbs enclosed in the product. The reported unrelated side effects include nervousness, insomnia (one participant reported not being able to sleep for three successive days), headache, palpitations, and hypertension. This observation raises the possibility of the presence of adulteration. A detailed supposed correlation between these adulterants and COVID-19 infection severity is illustrated in a S2 File.

### 4.4. Non-FDA-approved AOPs&COVID-19

The majority of participants in the current study (81%) consumed non-approved single- or multi-ingredient AOPs without medical consultation. The most used products were: metformin, green coffee, cinnamon, garcinia/chromium, Coffea-Arabica, African-mango, and Gymnema-Sylvestre.

#### 4.4.1. Metformin

Long-term follow-up from the Diabetes Prevention Program demonstrates that metformin induces weight loss. Many mechanisms can explain metformin-induced weight loss including a reduction in insulin secretion and hepatic gluconeogenesis, appetite suppression through modulation of hypothalamic appetite regulatory centers, alteration in the gut microbiome, changing the circadian rhythm, and regulation of fat oxidation and storage in different tissues [26]. In the current survey, a relatively large percentage of eligible participants were on metformin. About 43%of them reported an incidence of side-effects mainly hypoglycemia as expected from administering an antidiabetic agent to normoglycemic individuals, in addition to the well-documented metformin-induced GIT side-effects [27].

All participants who got infected with SARS-CoV-2 during metformin administration (71%of total participants on metformin) reported experiencing moderate-to-severe COVID-19 symptoms. Reports indicate the protective effect of metformin, as it can inhibit viral entry, multiplication, and translation of viral proteins besides its ability to modulate inflammation and immune responses in diabetic patients infected with COVID-19 [27]. Despite that, it is not clear whether this protective effect can be accomplished in normoglycemic subjects. A great percentage of participants in our study reported experiencing hypoglycemia when using metformin for weight reduction. COVID-19 can also cause hypoglycemia which contributes to the severity of COVID-19 symptoms [28]. Combined COVID-19- and metformin-induced hypoglycemias may explain the absence of cases with mild symptoms, as all participants on metformin reported moderate-to-severe infections.

The majority of eligible participants who used metformin for weight reduction continued using metformin after recovery from COVID-19. Nearly 30% of them reported decreased efficacy and 20% reported an increase in the incidence of hypoglycemia, especially in those suffering severe COVID-19 symptoms. Failure of the endocrine beta-cells during severe COVID-19-infection has been discussed by Shirakawa [29]. It was suggested that SARS-CoV-2 induces beta-cell apoptosis and decreases insulin secretion. The virus most probably infects beta-cells through neuropilin-1 and not ACE2 or transmembrane serine-protease-2(TMPRSS2) due to the observed low expression of ACE2 and TMPRSS2 on beta-cells of the endocrine pancreas [29]. Decreased functioning pancreatic beta-cells would certainly affect metformin efficacy following severe COVID-19 infections (Fig 3).

#### 4.4.2. Green-coffe

Green coffee is raw unroasted coffee beans that are a popular weight-loss supplement. The main weight-reducing ingredients are chlorogenic acids that act independently of caffeine to improve glucose metabolism. They decrease glucose uptake in the small intestine. They also inhibit the glucose-6-phosphatase system, amylolytic enzymes and sustain glucose homeostasis [30]. Green coffee bean extract has an excellent safety profile. However, green coffee does contain caffeine, which can cause tachycardia, frequent urination, insomnia, and anxiety. Accordingly, in the current questionnaire, most participants denied the incidence of side effects, and only 11.11%of participants administering green coffee reported the incidence of headache as a main side-effect.

Since chlorogenic acids inhibit glucose production which is the primary promoter for viral replication and inflammatory response, it is therefore expected that green-coffee administration would result in mild COVID-19 symptoms. However, in our survey, 50% of the patients reported experiencing severe symptoms. Strikingly, those experiencing severe symptoms are extremely obese with a BMI range of 35.6–47.8, indicating that those severe symptoms may be a consequence of obesity complications and not due to green coffee.

SARS-CoV-2 has mechanisms that ensure rapid and efficient replication and spread; It alters the host cells’ metabolism, such as peripheral blood monocytes, and enhances nutrient uptake including glucose to support aerobic-glycolysis. Consequently, these cells become highly glycolytic. In addition, COVID-19 infection can promote glycolysis through the generation of mitochondrial reactive oxygen specieshttps://www.sciencedirect.com/topics/medicine-and-dentistry/reactive-oxygen-species, which induces stabilization of hypoxia-inducible factor-1α [31]. Since this viral infection sustains high glucose levels in the host, this certainly would have a negative impact on the efficacy of green coffee which acts primarily by maintaining glucose homeostasis. This matches our survey results where 50%of patients continuing regular green coffee intake post-COVID-19-infection reported diminished activity in weight reduction, especially those experiencing severe infections.

#### 4.4.3. Cinnamo

Cinnamon extracts have been widely used in traditional medicine around the globe. Cinnamon consists of a variety of resinous compounds and essential oils [32]. Among the broad range of cinnamon therapeutic effects, it has been proven to have weight-loss effects through various mechanisms; it can reduce lipid accumulation in fat cells and adipose tissues by transcriptional regulation of CCAAT/enhancer-binding proteins and peroxisome-proliferator-activated-receptor-gamma (PPAR-γ). It can also modulate lipogenesis-related enzyme activity, such as fatty acid-synthase,acyl-CoA-carboxylase, and glycerol-3-phosphate-dehydrogenase. In addition, it can stimulate adipose tissue thermogenesis. Furthermore, cinnamon has the potential to augment insulin sensitivity and glucose transport. All these effects result in significant weight reduction experimentally and clinically [33].

Cinnamon and its ingredients have been recommended for improving COVID-19 infection due to its anti-obstructive, organ-protective, anti-inflammatory, anti-viral, and antioxidant effects. Cinnamon can also prevent re-infection through DNA-dependent enhancement of immunologic memory. All these actions are mostly attributed to cinnamon-induced PPAR-γ activation property [34]. In our survey, nearly 62%of participants on cinnamon experienced mild-to-moderate COVID-19 symptoms and those experiencing severe symptoms were obese with a BMI range of 31.4–41.7. This observation is common with most of the administered AOPs, suggesting the great negative impact of obesity on COVID-19 symptom relief.

Inflammatory conditions, such as in the case of COVID-19-infection, are coupled with overproduction of inflammatory cytokines and diminished PPAR-γ receptors [35]. Since PPAR-γ is one of the main molecular targets of cinnamon, diminished PPAR-γ receptors during COVID-19 infection may negatively affect cinnamon’s anti-obesity effects. However, only 15.4% of participants reported diminished activity post-COVID-19 infection. Possibly, other mechanisms by which cinnamon can reduce body weight compensated for the decreased level of PPAR-γ and maintained efficacy in weight loss in the majority of the patients.

#### 4.4.4. Garcinia-cambogia

*Garcinia-cambogia* is a herbal product that was traditionally used as a flavoring agent and has recently been used in herbal AOPs due to appetite suppressant properties. Garcinia extracts contain xanthones, benzophenones, amino acids, and the most biologically active ingredient is hydroxycitric-acid (HCA) the suspected ingredient responsible for its anti-inflammatory and appetite suppressant activity [35]. HCA can induce weight reduction through various mechanisms, the most important of which is controlling appetite via serotonin regulation and food intake suppression. HCA can increase serotonin release or availability in the brain and prevent the reuptake of released serotonin in brain neurons. In addition, HCA affects lipid metabolism as it decreases de novo lipogenesis by inhibiting ATP citrate lyase; the enzyme that catalyzes citrate cleavage into oxaloacetate and acetyl-coenzyme-A, and increases fatty acid oxidation. Besides, HCA can reduce plasma insulin and leptin levels together with tissue glucose intake leading to increased energy expenditure [36].

In the present study, only 10%of participants taking *Garcinia-cambogia* reported an incidence of diarrhea. In accordance, there are multiple reports of GIT problems in some people who have taken AOPs containing garcinia [37]. These include reported nausea, elevations in serum aminotransferase levels, and jaundice in individuals taking garcinia alone or in multi-ingredient supplements [37].

Since *Garcinia-cambogia* is proven to have an anti-inflammatory effect and immune-boosting activities, it is expected to have a protective effect against viral infection and to reduce the severity of infection. This is consistent with the observed results of our study where patients infected with COVID-19 and were receiving pure Garcinia experienced only mild-to-moderate symptoms confirming the protective effect of the herb on this viral infection.

Following SARS-CoV-2 host cell entry, there is upregulation of RAS components including Ang-II. It was previously proved that Ang-II is involved in the regulation of serotonin synthesis and release from serotonergic neuron terminals in a biphasic manner; at low Ang-II levels, there is decreased serotonin release, and at high Ang-II levels, there is increased serotonin release [38]. This means that serotonin levels during COVID-19 infection depend on SARS-CoV-2 titer. In other words, if the infection is mild, there is a slight increase in Ang-II and thus low brain serotonin levels with subsequent appetite stimulant effect counteracting the action of HCA and an apparent decrease in *Garcinia-cambogia* efficacy with subsequent weight gain. While, if the infection is severe, there is a significant increase in Ang-II level that causes increased serotonin level and appetite suppressant effect which potentiates *Garcinia-cambogia’s* weight-reducing effect. Since in our study, patients administering pure *Garcinia-cambogia* suffered from mild-to-moderate infection, thus, it is expected to observe decreased garcinia efficacy in those with moderate infection and no change in garcinia efficacy in those with mild symptoms. According to our data, 50%of patients reported decreased garcinia efficacy and 50% reported no change in garcinia efficacy which is a perfect match to the above explanation.

#### 4.4.5. Gymnema-Sylvestre

*Gymnema-Sylvestre* (*G*.*Sylvestre*) is traditionally used for its anti-diabetic and weight-reduction properties. The main active constituents of the plant are a group of acids termed gymnemic acids in addition to triterpene saponins. *Gymnema-Sylvestre* exerts anti-diabetic and weight reduction effects mainly via delaying glucose absorption. This is because gymnemic acid molecules and glucose molecules share similar atomic arrangements and can occupy the same locations on sugar receptors. The binding of gymnemic acid to these receptors would prevent their activation by orally-administered glucose molecules, thus, diminishing the sugar craving. In addition, gymnemic acid molecules can occupy glucose receptors in the absorptive intestinal layers, thereby, limiting intestinal glucose absorption, which results in low blood glucose levels. In addition, *G*.*Sylvestre* increases glucose utilization of glucose; as it is shown to increase the activities of enzymes responsible for utilization of glucose by increasing insulin release from pancreatic islet cells [39].

*G*.*Sylvestre* administration may have some benefits during COVID-19 infection; it can stimulate insulin secretion from the pancreas and can overcome insulin resistance, counteracting SARS-CoV-2-induced insulin resistance secondary to insulin-secreting pancreatic beta-cell damage.

Both SARS-CoV-2 and *G*.*Sylvestre* can cause loss of sweet taste sensation but in different mechanisms; SARS-CoV-2 weakens the olfactory sensory neurons that detect odors which in turn affects gustatory function [40]. On the other hand, gymnemic acid molecules can block glucose receptors located on taste buds leading to decreased sugar craving. Thus, patients may not feel the difference in *G*.*Sylvestre* activity during COVID-19 infection as reported by patients enclosed in our study. However, there is a key difference between taste sensation loss during COVID-19-infection and *G*.*Sylvestre* use, that is, after patients stop taking *G*.*Sylvestre*, they will crave sugar again with a total recovery of sweet taste sensation, while post-COVID-19 patients may suffer from ageusia that may last for months [40]. Therefore, the reported maintenance of *G*.*Sylvestre* efficacy post-COVID-19-infection may be attributable to the interference of SARS-CoV-2 with *G*.*Sylvestre* mechanisms and the reduced sugar craving post-COVID-19-infection may be due to post-COVID-19 ageusia and not due to the drug itself.

## 5. Conclusion

In most cases,the use of AOPs before or during COVID-19 infection resulted in beneficial outcomes, due to their anti-inflammatory and anti-viral replication effects, and patients experienced mild-to-moderate COVID-19 symptoms. Noticeably, those experiencing severe symptoms were extremely obese (BMI≥35), indicating that those severe symptoms may be due to obesity complications and not due to the AOP. Furthermore, SARS-CoV-2 interferes with the mechanism of action of almost all AOPs affecting their efficacy and incidence of side-effects, especially in extremely obese individuals. These mechanisms were thoroughly appraised in the current investigation. Therefore, any drug or supplement concurrently taken in the presence of SARS-CoV-2 must be preceded by medical consultation to avoid any undesired consequences.

## Supporting information

S1 Table Comparison between the top-administered AOPs according to severity of COVID-19 infection during AOPs intake. χ^2^: Chi-square test, MC: Monte Carlo. p: p-value for comparing the studied groups (Significant level at p ≤ 0.05). Numbers in the same row carrying the same alphabetical letters have no statistically significant difference between them.
(DOCX)

S2 Table Comparison between the top-administered AOPs according to AOPs efficacy post-COVID-19 infection. χ^2^: Chi-square test, MC: Monte Carlo. p: p-value for comparing the studied groups (Significant level at p ≤ 0.05). Numbers in the same row carrying the same alphabetical letters have no statistically significant difference between them.
(DOCX)

S3 Table Comparison between the different studied groups according to change in AOP side effects severity post-COVID-19 infection. χ^2^: Chi-square test, MC: Monte Carlo. p: p-value for comparing the studied groups (Significant level at p ≤ 0.05). Numbers in the same row carrying the same alphabetical letters have no statistically significant difference between them.
(DOCX)

S1 File The developed questionnaire in the present study. (PDF)

S2 File Supposed correlation between AOPs adulterants and COVID-19-infection severity. (DOCX)

S3 File Raw data used in the present study. (XLSX)

## References

[1] Popkin BM, Du S, Green WD, Beck MA, Algaith T, Herbst CH, et al. Individuals with obesity and COVID‐19: a global perspective on the epidemiology and biological relationships. Obesity reviews. 2020;21(11):e13128. doi: 10.1111/obr.13128 32845580 PMC7461480

[2] Schwartz MW, Seeley RJ, Zeltser LM, Drewnowski A, Ravussin E, Redman LM, et al. Obesity pathogenesis: an endocrine society scientific statement. Endocrine reviews. 2017;38(4):267–96. doi: 10.1210/er.2017-00111 28898979 PMC5546881

[3] Lim S, Shin SM, Nam GE, Jung CH, Koo BK. Proper management of people with obesity during the COVID-19 pandemic. Journal of obesity & metabolic syndrome. 2020 Jun 6;29(2):84. 32544885 10.7570/jomes20056 PMC7338495

[4] de Leeuw AJ, Oude Luttikhuis MA, Wellen AC, Müller C, Calkhoven CF. Obesity and its impact on COVID-19. Journal of Molecular Medicine. 2021 Jul;99(7):899–915. doi: 10.1007/s00109-021-02072-4 33824998 PMC8023779

[5] Young MJ, Clyne CD, Chapman KE. Endocrine aspects of ACE2 regulation: RAAS, steroid hormones and SARS-CoV-2. Journal of Endocrinology. 2020;247(2):R45–62. doi: 10.1530/JOE-20-0260 32966970

[6] Sallis R, Young DR, Tartof SY, Sallis JF, Sall J, Li Q, et al. Physical inactivity is associated with a higher risk for severe COVID-19 outcomes: a study in 48 440 adult patients. British journal of sports medicine. 2021;55(19):1099–105. doi: 10.1136/bjsports-2021-104080 33849909

[7] Al Heialy S, Hachim MY, Senok A, Gaudet M, Abou Tayoun A, Hamoudi R, et al. Regulation of angiotensin-converting enzyme 2 in obesity: implications for COVID-19. Frontiers in physiology. 2020;11:555039. doi: 10.3389/fphys.2020.555039 33071815 PMC7531362

[8] Gomez G, Stanford FC. US health policy and prescription drug coverage of FDA-approved medications for the treatment of obesity. International Journal of Obesity. 2018;42(3):495–500. doi: 10.1038/ijo.2017.287 29151591 PMC6082126

[9] Aldewachi H, Mustafa YF, Najm R, Ammar F. Adulteration of slimming products and its detection methods. Systematic Reviews in Pharmacy. 2020;11(3):289.

[10] De la Rosa A, Ghusn W, Sacoto D, Campos A, Cifuentes L, Feris F, et al. A comparison between weight loss outcomes with anti-obesity medications before and during Covid-19 pandemic at a tertiary weight management center. Obesity Pillars. 2022;4:100046. doi: 10.1016/j.obpill.2022.100046 37990666 PMC9714128

[11] Charan J, Biswas T. How to calculate sample size for different study designs in medical research?. Indian journal of psychological medicine. 2013;35(2):121–6. doi: 10.4103/0253-7176.116232 24049221 PMC3775042

[12] Olson K. An examination of questionnaire evaluation by expert reviewers. Field methods. 2010 Nov;22(4):295–318.

[13] Wang H, Wang L, Cheng Y, Xia Z, Liao Y, Cao J. Efficacy of orlistat in non-alcoholic fatty liver disease: A systematic review and meta-analysis. Biomedical reports. 2018;9(1):90–6. doi: 10.3892/br.2018.1100 29930810 PMC6007047

[14] Chu J, Xing C, Du Y, Duan T, Liu S, Zhang P, et al. Pharmacological inhibition of fatty acid synthesis blocks SARS-CoV-2 replication. Nature metabolism. 2021;3(11):1466–75. doi: 10.1038/s42255-021-00479-4 34580494 PMC8475461

[15] Yang JK, Lin SS, Ji XJ, Guo LM. Binding of SARS coronavirus to its receptor damages islets and causes acute diabetes. Acta diabetologica. 2010;47:193–9. doi: 10.1007/s00592-009-0109-4 19333547 PMC7088164

[16] Memon B, Abdelalim EM. ACE2 function in the pancreatic islet: implications for relationship between SARS‐CoV‐2 and diabetes. Acta Physiologica. 2021;233(4):e13733. doi: 10.1111/apha.13733 34561952 PMC8646749

[17] Holmes L Jr, Enwere M, Williams J, Ogundele B, Chavan P, Piccoli T, et al. Black–White risk differentials in COVID-19 (SARS-COV2) transmission, mortality and case fatality in the United States: translational epidemiologic perspective and challenges. International journal of environmental research and public health. 2020;17(12):4322. doi: 10.3390/ijerph17124322 32560363 PMC7345143

[18] Lee YS, Jun HS. Anti-diabetic actions of glucagon-like peptide-1 on pancreatic beta-cells. Metabolism. 2014;63(1):9–19. doi: 10.1016/j.metabol.2013.09.010 24140094

[19] Ronveaux CC, Tomé D, Raybould HE. Glucagon-like peptide 1 interacts with ghrelin and leptin to regulate glucose metabolism and food intake through vagal afferent neuron signaling. The Journal of Nutrition. 2015;145(4):672–80. doi: 10.3945/jn.114.206029 25833771 PMC4381768

[20] Ng SY, Wilding JP. Liraglutide in the treatment of obesity. Expert Opinion on Biological Therapy. 2014;14(8):1215–24. doi: 10.1517/14712598.2014.925870 24905058

[21] Ladenheim EE. Liraglutide and obesity: a review of the data so far. Drug design, development and therapy. 2015:1867–75. doi: 10.2147/DDDT.S58459 25848222 PMC4386791

[22] Jin T, Liu M. Comment on GLP-1-based drugs and COVID-19 treatment. Acta Pharmaceutica Sinica. B. 2020;10(7):1249. 32834951 10.1016/j.apsb.2020.05.006 PMC7255283

[23] Monda VM, Porcellati F, Strollo F, Gentile S. ACE2 and SARS-CoV-2 infection: might GLP-1 receptor agonists play a role?. Diabetes Therapy. 2020;11:1909–14. doi: 10.1007/s13300-020-00898-8 32749644 PMC7400747

[24] Fandiño J, Toba L, González-Matías LC, Diz-Chaves Y, Mallo F. GLP-1 receptor agonist ameliorates experimental lung fibrosis. Scientific Reports. 2020;10(1):18091. doi: 10.1038/s41598-020-74912-1 33093510 PMC7581713

[25] Pais R, Rievaj J, Larraufie P, Gribble F, Reimann F. Angiotensin II type 1 receptor-dependent GLP-1 and PYY secretion in mice and humans. Endocrinology. 2016;157(10):3821–31. doi: 10.1210/en.2016-1384 27447725 PMC5045501

[26] Yerevanian A, Soukas AA. Metformin: mechanisms in human obesity and weight loss. Current obesity reports. 2019 Jun 1;8:156–64. doi: 10.1007/s13679-019-00335-3 30874963 PMC6520185

[27] Scheen AJ. Metformin and COVID-19: from cellular mechanisms to reduced mortality. Diabetes & metabolism. 2020;46(6):423–6. doi: 10.1016/j.diabet.2020.07.006 32750451 PMC7395819

[28] Shah K, Tiwaskar M, Chawla P, Kale M, Deshmane R, Sowani A. Hypoglycemia at the time of Covid-19 pandemic. Diabetes & Metabolic Syndrome: Clinical Research & Reviews. 2020;14(5):1143–6. doi: 10.1016/j.dsx.2020.07.003 32668399 PMC7347476

[29] Shirakawa J. Pancreatic β‐cell fate in subjects with COVID‐19. Journal of Diabetes Investigation. 2021;12(12):2126. 34529355 10.1111/jdi.13671 PMC8668054

[30] Hu GL, Wang X, Zhang L, Qiu MH. The sources and mechanisms of bioactive ingredients in coffee. Food & Function. 2019;10(6):3113–26. doi: 10.1039/c9fo00288j 31166336

[31] de Las Heras N, Martín Giménez VM, Ferder L, Manucha W, Lahera V. Implications of oxidative stress and potential role of mitochondrial dysfunction in COVID-19: therapeutic effects of vitamin D. Antioxidants. 2020;9(9):897. doi: 10.3390/antiox9090897 32967329 PMC7555731

[32] Jakhetia V, Patel R, Khatri P, Pahuja N, Garg S, Pandey A, et al. Cinnamon: a pharmacological review. Journal of advanced scientific research. 2010;1(02):19–23.

[33] Lu M, Cao Y, Xiao J, Song M, Ho CT. Molecular mechanisms of the anti-obesity effect of bioactive ingredients in common spices: a review. Food & function. 2018;9(9):4569–81. doi: 10.1039/c8fo01349g 30168574

[34] Yakhchali M, Taghipour Z, Ardakani MM, Vaghasloo MA, Vazirian M, Sadrai S. Cinnamon and its possible impact on COVID-19: The viewpoint of traditional and conventional medicine. Biomedicine & Pharmacotherapy. 2021;143:112221. doi: 10.1016/j.biopha.2021.112221 34563952 PMC8452493

[35] Hirawat R, Saifi MA, Godugu C. Targeting inflammatory cytokine storm to fight against COVID-19 associated severe complications. Life sciences. 2021;267:118923. doi: 10.1016/j.lfs.2020.118923 33358906 PMC7831473

[36] Semwal RB, Semwal DK, Vermaak I, Viljoen A. A comprehensive scientific overview of Garcinia cambogia. Fitoterapia. 2015;102:134–48. doi: 10.1016/j.fitote.2015.02.012 25732350

[37] Vuppalanchi R, Bonkovsky HL, Ahmad J, Barnhart H, Durazo F, Fontana RJ, et al. Garcinia cambogia, either alone or in combination with green tea, causes moderate to severe liver injury. Clinical Gastroenterology and Hepatology. 2022;20(6):e1416–25. doi: 10.1016/j.cgh.2021.08.015 34400337 PMC9004424

[38] Nahmod VE, Finkielman S, Benarroch EE, Pirola CJ. Angiotensin regulates release and synthesis of serotonin in brain. Science. 1978;202(4372):1091–3. doi: 10.1126/science.152460 152460

[39] Pothuraju R, Sharma RK, Chagalamarri J, Jangra S, Kumar Kavadi P. A systematic review of Gymnema sylvestre in obesity and diabetes management. Journal of the Science of Food and Agriculture. 2014;94(5):834–40. doi: 10.1002/jsfa.6458 24166097

[40] Risso D, Drayna D, Morini G. Alteration, reduction and taste loss: main causes and potential implications on dietary habits. Nutrients. 2020;12(11):3284. doi: 10.3390/nu12113284 33120898 PMC7693910

