# Supplementary material for: Cross-relationship between COVID-19 infection and anti-obesity products efficacy and incidence of side effects: A cross-sectional study
Source: PLoS One. 2024 Aug 22;19(8):e0309323. doi: 10.1371/journal.pone.0309323 (PMC11341056; doi:10.1371/journal.pone.0309323)
Supplement: S1 Table — χ2: Chi-square test, MC: Monte Carlo. p: p-value for comparing the studied groups (Significant level at p ≤ 0.05). Numbers in the same row carrying the same alphabetical letters have no statistically significant difference between them. (DOCX) [file pone.0309323.s001.docx]

**Supplementary tables**

**Table (S1): Comparison between the top-administered AOPs according to severity of COVID-19 infection during AOPs intake**

| **The severity of COVID-19 infection during AOPs intake** | **Drugs** | | | | | | |
| --- | --- | --- | --- | --- | --- | --- | --- |
|  | **Orlistat (n = 24)** | **Liraglutide (n = 24)** | **Metformin (n = 20)** | **Green coffee (n = 12)** | **Cinnamon (n = 12)** | ***Garcinia cambogia* (n = 6)** | ***Gymnema Sylvestre* (n = 2)** |
| Mild | 6^a^ (25.0%)) | 4^ab^ (16.7%) | 0^b^ (0.0%) | 3^a^ (25.0%) | 4^a^ (33.3%) | 1^ab^ (16.7%) | 2^c^ (100.0%) |
| Moderate | 13^abcde^ (54.2%) | 10^abcde^ (41.7%) | 12^abcde^ (60.0%) | 3^de^ (25.0%) | 4^ce^ (33.3%) | 5^b^ (83.3%) | 0^acde^ (0.0%) |
| Severe | 5^a^ (20.8%) | 10^ab^ (41.7%) | 8^ab^ (40.0%) | 6^b^ (50.0%) | 4^ab^ (33.3%) | 0^a^ (0.0%) | 0^a^ (0.0%) |
| **χ^2^** | 21.321^*^ | | | | | | |
| **^MC^p** | 0.022^*^ | | | | | | |

χ^2^: **Chi-square test** MC: **Monte Carlo**

p: p-value for comparing the studied groups (Significant level at p ≤ 0.05).

Numbers in the same row carrying the same alphabetical letters have no statistically significant difference between them.
