# Supplementary material for: Cross-relationship between COVID-19 infection and anti-obesity products efficacy and incidence of side effects: A cross-sectional study
Source: PLoS One. 2024 Aug 22;19(8):e0309323. doi: 10.1371/journal.pone.0309323 (PMC11341056; doi:10.1371/journal.pone.0309323)
Supplement: S2 Table — χ2: Chi-square test, MC: Monte Carlo. p: p-value for comparing the studied groups (Significant level at p ≤ 0.05). Numbers in the same row carrying the same alphabetical letters have no statistically significant difference between them. (DOCX) [file pone.0309323.s002.docx]

**Table (S2): Comparison between the top-administered AOPs according to AOPs efficacy post-COVID-19 infection**

| **Efficacy of AOPs post-COVID-19 infection** | **Drugs** | | | | | | |
| --- | --- | --- | --- | --- | --- | --- | --- |
|  | **Orlistat (n = 24)** | **Liraglutide (n = 24)** | **Metformin (n = 20)** | **Green coffee (n = 11)** | **Cinnamon (n = 12)** | ***Garcinia cambogia* (n = 6)** | ***Gymnema Sylvestre* (n = 2)** |
| Decreased | 9^a^ (37.5%) | 0^b^ (0.0%) | 6^a^ (30.0%) | 6^a^ (54.5%) | 2^a^ (16.7%) | 3^a^ (50.0%) | 0^ab^ (0.0%) |
| Remained the same | 15^a^ (62.5%) | 24^b^ (100.0%) | 14^a^ (70.0%) | 5^a^ (45.5%) | 10^a^ (83.3%) | 3^a^ (50.0%) | 2^ab^ (100.0%) |
| **χ^2^** | 19.966^*^ | | | | | | |
| **^MC^p** | 0.001^*^ | | | | | | |

χ^2^: **Chi-square test** MC: **Monte Carlo**

p: p-value for comparing the studied groups (Significant level at p ≤ 0.05).

Numbers in the same row carrying the same alphabetical letters have no statistically significant difference between them
