# Supplementary material for: Cross-relationship between COVID-19 infection and anti-obesity products efficacy and incidence of side effects: A cross-sectional study
Source: PLoS One. 2024 Aug 22;19(8):e0309323. doi: 10.1371/journal.pone.0309323 (PMC11341056; doi:10.1371/journal.pone.0309323)
Supplement: S3 Table — χ2: Chi-square test, MC: Monte Carlo. p: p-value for comparing the studied groups (Significant level at p ≤ 0.05). Numbers in the same row carrying the same alphabetical letters have no statistically significant difference between them. (DOCX) [file pone.0309323.s003.docx]

**Table (S3): Comparison between the different studied groups according to change in AOP side effects severity post-COVID-19 infection**

| **Change in AOPs side effects severity post-COVID-19 infection** | **Drugs** | | | | | | |
| --- | --- | --- | --- | --- | --- | --- | --- |
|  | **Orlistat (n = 24)** | **Liraglutide (n = 24)** | **Metformin (n = 20)** | **Green coffee (n = 11)** | **Cinnamon (n = 12)** | ***Garcinia cambogia* (n = 6)** | ***Gymnema Sylvestre* (n = 2)** |
| No change | 22^ab^ (91.7%) | 24 ^a^ (100.0%) | 16^ab^ (80.0%) | 11^a^ (100.0%) | 11^ab^ (91.7%) | 6^a^ (100.0%) | 2^a^ (100.0%) |
| More side effects | 2^a^ (8.3%) | 0^a^ (0.0%) | 4^ab^ (20.0%) | 0 ^a^ (0.0%) | 1^a^ (8.3%) | 0^a^ (0.0%) | 0^a^ (0.0%) |
| **χ^2^** | 6.849 | | | | | | |
| **^MC^p** | 0.240 | | | | | | |

χ^2^: **Chi-square test** MC: **Monte Carlo**

p: p-value for comparing the studied groups (Significant level at p ≤ 0.05).

Numbers in the same row carrying the same alphabetical letters have no statistically significant difference between them
