# Supplementary material for: Cross-relationship between COVID-19 infection and anti-obesity products efficacy and incidence of side effects: A cross-sectional study
Source: PLoS One. 2024 Aug 22;19(8):e0309323. doi: 10.1371/journal.pone.0309323 (PMC11341056; doi:10.1371/journal.pone.0309323)
Supplement: S1 File — (PDF) [file pone.0309323.s004.pdf]

أدوية التخسيس: فاعليتها وآثارها الجانبية

* Indicates required question

بيانات خاصة بالمريض

1. النوع *

Mark only one oval.

- أنثى
- ذكر

2. العمر *

Mark only one oval.

- 15-20
- 20-30
- 30-40
- 40-50
- 50<

3. الوزن الحالي *

4. الطول *

5. المشاكل الطبية المزمنة

Check all that apply.

- [ ] سكر
- [ ] ارتفاع ضغط الدم
- [ ] امراض جهاز تنفسي
- [ ] حساسية
- [ ] كوليسترول ودهون ثلاثية
- [ ] اخرى
- [ ] لا يوجد مشاكل طبية

6. إذا كانت لديك مشكلة طبية مزمنة أخرى، فما هي؟

بيانات خاصة بدواء التخسيس الذي تم استخدامه

7. نوع الدواء *

Check all that apply.

- [ ] دواء طبي
- [ ] منتج طبيعي

المنتجات الطبيعية

8. اسم الدواء *

9. الهدف من تناول الدواء *

Check all that apply.

- [ ] زياده معدل الحرق
- [ ] زياده الإحساس الشبع
- [ ] تقليل امتصاص الدهون
- [ ] التأثير على مركز الجوع بالمخ
- [ ] أخرى

10. هل ساعد الدواء في انقاص الوزن ؟ *

Mark only one oval.

- ( ) نعم
- ( ) لا

11. مقدار خسارة الوزن في الأسبوع (كجم) *

____

12. هل تسبب الدواء في حدوث أي آثار جانبية؟ *

Mark only one oval.

- ( ) نعم
- ( ) لا

13. ما هي الأثار الجانبية التي واجهتها؟ *

____

14. هل تتبع نظامًا غذائيًا أثناء تناول الدواء؟

Mark only one oval.

- ( ) نعم
- ( ) لا

15. ما نوع النظام الغذائي الذي تتبعه؟ مثال (كيتو، لقيمات، عجز في السعرات الحرارية, صيام متقطع، نظام منخفض الكربوهيدرات)

____

16. هل سبق لك الإصابة بفيروس كورونا أثناء تناول الدواء؟ *

Mark only one oval.

- ( ) نعم
- ( ) لا

17. كيف كانت حدة الأعراض ؟

Mark only one oval.

- ( ) شديدة
- ( ) متوسطة
- ( ) خفيفة

18. * هل كنت منتظمًا على تناول الدواء بعد الاصابة بعدوي كورونا؟

Mark only one oval.

- نعم
- احيانًا
- لا

19. * تأثير الدواء بعد الإصابة بفيروس كورونا؟

Mark only one oval.

- قل تأثيره
- زاد تأثيره
- ظل ثابتًا

20. * هل ظهرت أي أعراض جانبية جديدة أثناء استخدام دواء التخسيس مع بروتوكول علاج فيروس كورونا؟

Mark only one oval.

- نعم
- لا

21. إذا كانت إجابتك بنعم على السؤال السابق، فما هي تلك الأعراض؟

المنتجات الطبية

22. * اسم الدواء

23. * الهدف من تناول الدواء

Check all that apply.

- [ ] زيادة معدل الحرق
- [ ] زيادة الإحساس بالشبع
- [ ] تقليل امتصاص الدهون
- [ ] التأثير على مركز الجوع بالمخ
- [ ] أخرى

24. * هل ساعد الدواء في انقاص الوزن ؟

Mark only one oval.

- نعم
- لا

25. * مقدار خسارة الوزن في الاسبوع (كجم)

26. * هل تسبب الدواء في حدوث آثار جانبية؟

Mark only one oval.

- نعم
- لا

27. ما هي الآثار الجانبية التي واجهتها؟

28. هل تتبع نظامًا غذائيًا أثناء تناول الدواء؟ *

Mark only one oval.

- نعم
- لا

29. ما نوع النظام الغذائي الذي تتبعه؟ مثال [كيتو، لقيمات، عجز في السعرات الحرارية، نظام منخفض الكربوهيدرات]

____

30. هل سبق لك الإصابة بفيروس كورونا أثناء تناول الدواء؟ *

Mark only one oval.

- نعم
- لا

31. كيف كانت حدة الأعراض ؟

Mark only one oval.

- شديدة
- متوسطة
- خفيفة

32. هل كنت منتظمًا على تناول الدواء بعد الاصابة بعدوي كورونا؟ *

Mark only one oval.

- نعم
- احيانًا
- لا

33. تأثير الدواء بعد الإصابة بفيروس كورونا؟ *

Mark only one oval.

- قل تأثيره
- زاد تأثيره
- ظل ثابتًا

34. هل ظهرت أي أعراض جانبية جديدة أثناء استخدام دواء التخسيس مع بروتوكول علاج فيروس كورونا؟ *

Mark only one oval.

- نعم
- لا

35. إذا كانت إجابتك بنعم على السؤال السابق، فما هي تلك الأعراض؟

____

This content is neither created nor endorsed by Google.

Google Forms
