# Supplementary material for: Cross-relationship between COVID-19 infection and anti-obesity products efficacy and incidence of side effects: A cross-sectional study
Source: PLoS One. 2024 Aug 22;19(8):e0309323. doi: 10.1371/journal.pone.0309323 (PMC11341056; doi:10.1371/journal.pone.0309323)
Supplement: S2 File — (DOCX) [file pone.0309323.s005.docx]

**Supplementary** **S2 File.** **Supposed correlation between AOPs adulterants and COVID-19-infection severity**

The most reported undeclared ingredients, which may be illegally added include; sibutramine, amphetamines, phentermine, phenolphthalein, bupropion, bumetanide, and phenytoin in weight-reducing and fat-loss supplements (1). Although phentermine and bupropion are listed among the illegal adulterants, they belong to the FDA-approved AOPs when combined with topiramate and naltrexone, respectively.

**1. Topiramate/Phentermine**

Topiramate is an anti-epileptic drug that acts via several pharmacological mechanisms; primarily through enhancement of GABA-ergic activity, in addition to, inhibition of voltage-sensitive sodium and calcium channels, kainate/α-amino-3-hydroxy-5-methylisoxazole-4-proprionic acid-type glutamate receptors, high-frequency action potential firing and carbonic anhydrase. On the other hand, phentermine is a noradrenergic drug that affects food intake primarily via stimulating norepinephrine release with no serotonergic or dopaminergic activity at clinical doses. Unlike amphetamine, phentermine has low addiction potential (2).

Correlating the key molecular targets of topiramate and phentermine; GABA and norepinephrine, to COVID-19, may provide a clue on the expected outcome if these agents were administered before or post-COVID-19 infection.

SARS-CoV-2 brain invasion triggers reactive astrogliosis and activates the microglia to release myriad pro-inflammatory cytokines (TNF-α, IL-6, IL-1B), nitric oxide, prostaglandin E2, and free radicals in brain areas involved in epileptic pathogenesis. This may cause chronic inflammation and neural hyper-excitability triggering seizure incidence (3). Therefore, intake of topiramate before or during COVID-19 infection may decrease susceptibility to experience epileptic seizures which may add benefit to epilepsy sufferers when infected with SARS-CoV-2. However, since SARS-CoV-2 interferes with topiramate’s mechanism, the efficacy of this drug in weight reduction may be affected.

During COVID-19 infection, the increased levels of catecholamine lead to renin-angiotensin-aldosterone system (RAAS) activation which promotes SARS-CoV-2 entry and causes COVID-19 complications (4). Thus, adrenergic agonists would worsen the condition of COVID-19 patients and adrenergic blockers would decrease the SARS-CoV-2 virus cellular entry (5). Accordingly, phentermine as an adrenergic drug would exacerbate COVID-19 infection. In addition, the increased production of catecholamines during COVID-19 infection would exaggerate the incidence of phentermine-induced side effects.

**2. Naltrexone-bupropion**

This medication is mainly used by certain overweight people having weight-related medical problems. Naltrexone belongs to a class of drugs known as opiate antagonists (blocks the μ- and κ-opioid receptors), and bupropion is an antidepressant. These two medications work synergistically in the hypothalamus and the mesolimbic dopamine circuit to reduce appetite (6).

Combined naltrexone/bupropion preparation is suggested to protect against COVID-19 infection. It also can attenuate COVID-19 infection severity. Furthermore, this combined preparation was proposed as the first-line treatment option for obese patients infected with COVID-19 in 2022 (6). Naltrexone, especially at low doses, can inhibit viral entry and infectivity via interfering with SARS-CoV-2 Spike protein binding to ACE2 and diminishing hyper-inflammatory cytokines storm. These actions can be done through naltrexone-induced blockade of ERK1/2 phosphorylation and suppression of high fat/lipopolysaccharide-induced pro-inflammatory cytokine release both from macrophage cells and adipose tissue macrophage (7, 8). Moreover, Low Dose Naltrexone (LDN) may disrupt the interaction of ACE2 with the SARS-CoV-2 receptor binding domain as proved by virtual docking and simulation data (7). Furthermore, LDN can reduce COVID-19 immune-mediated thrombotic complications by blunting innate immune responses, attenuating Toll-like receptor signaling, and decreasing the levels of tumor necrosis factor-alpha, interferon, and interleukin-1 (8). The other member of this combined preparation; bupropion, was found also to add benefit in individuals infected with COVID-19. Bupropion can treat COVID–19–induced neurocognitive complications, such as brain fog, by increasing the neural activity in the cingulate cortex, decreasing proinflammatory cytokines, and blocking hippocampal cell loss (9).

The impact of COVID-19 infection on the efficacy of this combination is not clear, yet, it can be theoretically predicted; Naltrexone acts mainly as an antagonist on the mu-opioid receptor and it is a weaker antagonist of the kappa and delta-opioid receptors (10). During active COVID-19 infection, the Ang-II peptide level peaks (11). This peptide mediates its actions on the CNS via the binding of endogenous opioid agonists to kappa rather than mu-opioid receptors (12). This means that Ang-II has a weak effect on the molecular target of naltrexone (mu-opioid receptor). Consequently, the elevated Ang-II levels during COVID-19 infection could only minimally affect the efficacy of naltrexone in this combination. On the other hand, bupropion is a dopamine and norepinephrine reuptake inhibitor that increases the brain levels of these neurotransmitters (9). Since COVID-19 infection is coupled with increased levels of catecholamines, it is expected that the action and incidence of side effects of bupropion will increase during infection. In line with our expected impact of COVID-19 infection on combined naltrexone/bupropion preparation efficacy and incidence of side effects, patients reported worsening post-COVID-19 symptoms with this medication. These outcomes are predicted and are not supported by data from our study as no participant reported administration of this combination.

**References**

1. Aldewachi H, et al. Adulteration of slimming products and its detection methods. Syst Rev Pharm. 11(3):289 (2020).
2. Shin JH & Gadde KM. Clinical utility of phentermine/topiramate (Qsymia™) combination for the treatment of obesity. *Diabetes Metab. Syndr. Obes*. 131-9 (2013).
3. Nikbakht F, et al. How does the COVID-19 cause seizure and epilepsy in patients? The potential mechanisms. *Mult. Scler. Relat. Disord*. **46**:102535 (2020).
4. Alsagaff MY & Mulia EP. Hypertension and COVID-19: Potential use of beta-blockers and a call for randomized evidence. *IHJ.* **73**(6):757-9 (2021).
5. Vasanthakumar N. Beta‐adrenergic blockers as a potential treatment for COVID‐19 patients. BioEssays. **42**(11):2000094 (2020).
6. Sherman MM, et al. Naltrexone/bupropion ER (Contrave): newly approved treatment option for chronic weight management in obese adults. *Pharm. Ther*. **41**(3):164 (2016).
7. Choubey A, et al. Naltrexone a potential therapeutic candidate for COVID-19*. J. Biomol. Struct. Dyn*. **40**(3):963-70 (2022).
8. Pitt B, et al. Repurposing low-dose naltrexone for the prevention and treatment of immunothrombosis in COVID-19. *Eur. Heart J. Cardiovasc. Pharmacother*. **8**(4):402-5 (2022).
9. Reinfeld S. Can bupropion treat COVID-19–induced brain fog? A case series. *Int. Clin. Psychopharmacol.* **15**:10-97 (2022).
10. Niciu MJ & Arias AJ. Targeted opioid receptor antagonists in the treatment of alcohol use disorders. *CNS drugs*. **27**(10):777-87 (2013).
11. Xavier LL, et al. Does angiotensin II peak in response to SARS-CoV-2?. *Front. Immunol.* **11**:577875 (2021).
12. Rabkin SW. Endogenous kappa opioids mediate the action of brain angiotensin II to increase blood pressure. *Neuropeptides*. **41**(6):411-9 (2007).
